# Preferences for Salty and Sweet Tastes Are Elevated and Related to Each Other during Childhood

**DOI:** 10.1371/journal.pone.0092201

**Published:** 2014-03-17

**Authors:** Julie A. Mennella, Susana Finkbeiner, Sarah V. Lipchock, Liang-Dar Hwang, Danielle R. Reed

**Affiliations:** Monell Chemical Senses Center, Philadelphia, Pennsylvania, United States of America; German Institute for Human Nutrition, Germany

## Abstract

**Background:**

The present study aimed to determine if salty and sweet taste preferences in children are related to each other, to markers of growth, and to genetic differences.

**Methods:**

We conducted a 2-day, single-blind experimental study using the Monell two-series, forced-choice, paired-comparison tracking method to determine taste preferences. The volunteer sample consisted of a racially/ethnically diverse group of children, 5–10 years of age (n = 108), and their mothers (n = 83). After excluding those mothers who did not meet eligibility and children who did not understand or comply with study procedures, the final sample was 101 children and 76 adults. The main outcome measures were most preferred concentration of salt in broth and crackers; most preferred concentration of sucrose in water and jelly; reported dietary intake of salty and sweet foods; levels of a bone growth marker; anthropometric measurements such as height, weight, and percent body fat; and *TAS1R3* (sweet taste receptor) genotype.

**Results:**

Children preferred higher concentrations of salt in broth and sucrose in water than did adults, and for both groups, salty and sweet taste preferences were significantly and positively correlated. In children, preference measures were related to reported intake of sodium but not of added sugars. Children who were tall for their age preferred sweeter solutions than did those that were shorter and percent body fat was correlated with salt preference. In mothers but not in children, sweet preference correlated with *TAS1R3* genotype.

**Conclusions and Relevance:**

For children, sweet and salty taste preferences were positively correlated and related to some aspects of real-world food intake. Complying with recommendations to reduce added sugars and salt may be more difficult for some children, which emphasizes the need for new strategies to improve children's diets.

## Introduction

Many illnesses of modern society are, in part, the consequence of poor food choices. Although foods high in salt (NaCl) and refined sugars contribute to poor health [1], people of all ages consume them in excessive amounts [2], [3], [4], in part because these foods have powerful hedonic appeal, especially for children [5]. Current intakes of sodium and added sugars by the youngest members of our society are well in excess of recommended dietary intakes [2], [6]. Over 90% of American children 2–8 years of age are getting more than half of their discretionary calorie allowance from added sugars [7]. Likewise, sodium intake is approximately 3,200 mg/day (excluding table salt) [8], well above adequate levels of 1,200–1,500 mg/day for 4- to 13-year-olds [9].

To better understand food choices among children, the present study examined individual differences in preference for sugars and salt. It has been argued that evolutionarily driven taste preferences predispose children to consume such diets, with the liking of sweetness attracting them to mothers' milk and fruits, and the liking of salty taste attracting them to sodium and possibly other minerals needed for bone growth [10]. From this perspective, humans evolved a desire to consume once rare calorie- and sodium-rich foods [11], and children's basic biology programs them to like sweet sources of energy and salty minerals during periods of growth [12], [13]. These preferences are not necessarily related to overeating and obesity [14], although they might be under certain circumstances.

With these points in mind, we developed three predictions. First, individual differences in the liking of sodium chloride and sucrose should be concordant in children, as they are in adults [15], and should generalize to a variety of foods. Second, urinary levels of cross-linked N-telopeptides of type I collagen (NTx), a biomarker for bone resorption and growth that is higher during growth spurts [16] and has been linked to sweet liking [17], should also be linked to salty taste liking. Anthropometric measures such as height-for-age, weight, and percent body fat [18], which reflect growth differences among children [19], may also be related to taste preferences. Third, taste preferences may be affected by in-born differences in a sweet taste receptor gene [14], [20].

## Methods

### Participants

The sample included 108 healthy children 5–10 years of age (61 singletons, 19 sibling pairs, 3 sibling triads) and their mothers (n = 83), none of whom were taking prescription medications (except for birth control pills among women). Mothers were queried about race/ethnicity of themselves and their children, highest education level and family yearly income. Procedures were approved by the Office of Regulatory Affairs at the University of Pennsylvania. Before testing, written informed consent was obtained from each adult, and assent was obtained from each child ≥7 years of age.

### Stimuli

Following from psychophysical studies [5], [21], five sucrose solutions of varying concentrations (3–36% wt/vol) were used to determine most preferred level of sweetness, and five soup stimuli, which were made by adding varying levels of sodium (0.92–6.14% wt/vol NaCl) to a vegetable broth (Campbell Soup Co., Camden, NJ), were used to determine most preferred level of saltiness (primary outcomes). To validate real-world relevance of these primary measures, we assessed most preferred levels of sucrose and salt in jellies and crackers (secondary outcomes), respectively. Five grape jellies with differing sugar concentrations (30–70% wt/wt) were prepared by adding superfine sugar to low-sugar Concord grape jelly (J.M. Smucker Co., Orrville, OH; 30% w/w sugar content, containing no non-caloric sweeteners). As a reference, regular-sugar commercial jellies are ∼50–60% sugar wt/wt [22]. Three crackers that differed in salt content (0.80%, 2.90%, 6.12% dry wt/wt NaCl) were also prepared; the midpoint represents salt content of crackers on the market [22].

### Procedures

Following abstinence from eating for at least 1 hour, participants were tested individually on two separate days in rooms specifically designed for sensory testing. In counterbalanced order, we determined the concentration of sucrose and salt most preferred in water and broth on one testing day and of sucrose and salt most preferred in jellies and crackers on another. To reduce visual differences among samples, testing occurred in rooms illuminated with red lights.

#### Preference tests

Procedures were identical for both children and adults. The Monell two-series, forced-choice, paired-comparison tracking method [5], [21] was used to determine most preferred levels of salty taste in broth and sweet taste in solution. In brief, participants were presented with pairs of broth twice during one session and of sucrose solutions twice during another (5 ml each). They tasted each pair item for 5 s and then pointed to which they liked better, without instruction on how the stimuli differed. They rinsed their mouths between each sample and pair. Each subsequent pair contained the selected concentration paired with an adjacent stimulus concentration. This pattern continued until the participant either chose two consecutive times the same concentration paired with both a higher and lower concentration, or chose the highest or lowest concentration twice. The entire task was repeated after a 3-min break, with stimulus pairs presented in reverse order (i.e., weaker stimulus presented first in the first series; stronger stimulus first in the second series). This was done to ensure that children's responses were not due to choosing whatever came first (position bias). The geometric mean of the two concentrations chosen in series 1 and 2 provided an estimate for most preferred levels of sweet or salty tastes. Similar psychophysical testing methods were used to determine the most preferred level of salty tastes in crackers (15 mm diameter each) and most preferred level of sweet taste in jellies (2 g each).

#### Bone resorption marker and anthropometry

NTx levels in children's urine were measured by immunoassay using the Osteomark NTx Urine kit (Wampole Laboratories, Princeton, NJ) [23], [24]. NTx was chosen because it is a specific and sensitive marker for bone resorption and growth [16], with levels ranging from 112 to 1,619 nM bone collagen equivalents (BCE)/mM creatinine in children (5–10 years of age) [23], and because the decline in sweet preference that occurs during adolescence was previously shown to relate to decreases in urinary NTx [17]. Nanomolar concentrations of BCE were determined using a four-parameter curve-fitting equation in KaleidaGraph 3.6.4 (Synergy Software, Reading, PA). Samples were run in duplicate; the coefficient of variation averaged 6.7%. Urine samples were diluted 1∶5 with a known concentration of BCE following manufacturer instructions. The dilution factor and the background diluent BCE were incorporated into the final calculation using the equation *X*_sample_ = 5×[*X*_assay_−(0.8×*X*_diluent_)], where *X* is the concentration (nM) of BCE. For each sample, BCE of NTx immunoreactivity was normalized to creatinine [25].

Children and their mothers were weighed and measured for height wearing light clothing and no shoes (Detecto, Webb City, MO). For children, age- and sex-specific body mass index (BMI) *z*-scores were calculated using EpiInfo 3.5 (www.cdc.gov/epiinfo). Percent body fat was estimated by bioelectrical impedance analysis [26] using the Quantum X instrument (RJL Systems, Clinton Township, MI).

#### Dietary habits and demographics

Demographic data were collected by interview, and dietary intake data were collected and analyzed using the Automated Self-Administered 24-Hour Recall system, developed by the National Cancer Institute (Bethesda, MD) [27]. On each testing day, mothers and children sat side by side as the mother reported to a trained researcher 24-hour dietary recall for herself and then for her child. Children were also asked to report on snacks or foods eaten outside the home (e.g., school). From these data, we focused on daily sodium intake (mg Na/day), caloric intake (kcal/day), and added sugar intake (g added sugar/day) [28]. Values represent the average of the 2 days of diet reports.

#### Taste receptor genotyping

A variant of the *TAS1R3* sweet receptor gene, previously related to sweet preference and sensitivity [20], was genotyped (rs35744813; CC, CT, and TT). Subjects provided DNA samples extracted from cheek swabs or saliva (BuccalAmp, Epicenter, Madison, WI, or Genotek, Kanata, Canada). DNA samples were used as template in Taqman assays (Applied Biosystems, Foster City, CA).

### Statistical Analyses

We determined whether the primary outcome measures (most preferred concentration of sugar in water and salt in broth) differed by age group (children vs. mothers) using separate ANOVAs. For children, sex differences in the primary outcome measures were evaluated by t-tests. We also analyzed correlations between participants' most preferred concentrations of sucrose and salt and most preferred crackers varying in salt and jellies varying in sucrose (secondary outcomes), as well as reported dietary intakes of sodium and added sugars. For children, we examined correlations between primary measures of salty and sweet taste preference and NTx levels in urine, height, weight, and percent body fat. The correlations were done separately for boys and girls because growth patterns differ between sexes [29]. An additional method of analysis was conducted so these data could be directly compared with a similar study [17]: children were split at the median sucrose preference value, and again at the median salt preference value and the groups were compared for age, height, weight, percent body fat, and NTx levels in urine. For the genetics analysis, two-way ANOVA with age group (generation: mother vs. child) and sweet receptor genotype as the fixed factors was also conducted. For siblings, one child was picked randomly for analysis so that all children were unrelated. Prior to analysis, data were evaluated by the Kolmogorov-Smirnov test to determine if they were normally distributed, and the data were also examined for extreme values, which were removed from the analysis as noted in the Results Section. Analyses were conducted with Statistica 8 (StatSoft, Tulsa, OK); data are means ± SEM, with statistical significance at p≤0.05. Fisher's least squares difference tests were conducted on significant results to compare specific group means.

To explore the relative contribution of growth measures on salty and sweet taste preference, a multivariate general linear model was evaluated [30], which was constructed based on information from the univariate analysis and included NTx, height, weight, and percent body fat as predictor variables. The main dependent variables (preference for sucrose in water and preference for salt in broth) were correlated with one another, so both were used as outcome measures in multivariate Model 1, using sex and age as covariates. Models 2–4 were evaluated without outliers and separately for sweet and salty preferences, respectively.

## Results

### Completion of tasks

Seven children did not understand the psychophysical tasks or did not comply with study procedures, and seven mothers did not undergo psychophysical testing because they did not meet the inclusion criteria, thus resulting in a final participant sample of 101 children and 76 adults (**Table 1**), which reflected the diversity of race/ethnicity, family income, and educational levels of the city of Philadelphia [31]. Not all subjects completed all tasks (**Table 1**): most (95 children, 74 adults) provided complete dietary data; 99 children agreed to provide urine samples, and 80 provided samples that yielded valid NTx data (the other samples either were inadequate or yielded out-of-range values).

**Table 1:** Subject characteristics, dietary intake, and completion of psychophysical tasks by age group.

Measure	Age Group	
	Mothers (N = 76)	Children (N = 101)
Age, years [mean (SEM)]	36.1 (1.0)	7.8 (0.2)
Race/ethnicity [% (*n*)]		
White	32.9% (25)	31.7% (32)
Black	52.6% (40)	42.6% (43)
Hispanic/Latino/Latina	5.3% (4)	8.9% (9)
Asian	1.3% (1)	2.0% (2)
Other/more than one race	7.9% (6)	14.9% (15)
NTx/creatinine (nM BCE/mM; mean (SEM)]	—	506.34 (32.4)
BMI [kg/m^2^; mean (SEM)]	28.3 (7.0)	
Weight category by BMI [% (*n*)]^a^		
Underweight	2.6% (2)	5.9% (6)
Normal weight	32.9% (25)	64.4% (65)
Overweight	27.6% (21)	19.8% (20)
Obese	36.8% (28)	9.9% (10)
Height [m; mean (SEM)]	1.63 (0.01)	1.29 (0.01)
Percent body fat (%; mean (SEM)]	37.44 (0.84)	24.84 (0.99)
Dietary intake [mean (SEM)]^b^		
Energy intake, kcal/day	1882.1 (72.9)	1857.8 (63.6)
Added sugar		
g/day	78.6 g (5.8)	74.0 g (4.6)
teaspoons/day^c^	19.6 tsp (1.4)	18.5 tsp (1.2)
Sodium, mg/day	3246.6 (140.7)	3005.1 (122.7)
Socioeconomic data (adults only) [% (*n*)]		
Highest education level		
High school	23.7% (18)	
Some college/technical school	23.7% (18)	
College graduate	47.4% (36)	
Graduate school or higher	5.3% (4)	
Yearly income level^d^		
<$15,000	18.4% (14)	
$15,000–35,000	36.8% (28)	
$35,000–75,000	32.9% (25)	
>$75,000	10.5% (8)	
Subjects that completed psychophysical tasks (*n*)		
Most preferred level of sucrose in water	76	100
Most preferred level of sucrose in jelly	76	97
Most preferred level of salt in broth	75	96
Most preferred level of salt in crackers	76	98

a Categories to classify BMI are from the Centers for Disease Control and Prevention for children [44] and standard BMI categories for mothers.

b Intake data are averaged from the 2 testing days and were collected and analyzed using the Automated Self-Administered 24-Hour Recall, beta version (2009).

c One teaspoon of added sugar is 4 g; adapted from the National Cancer Institute.

d Income level is in US dollars; one mother did not report her income (n = 75).

### Effect of age and relationship with diet

**Figure 1:**
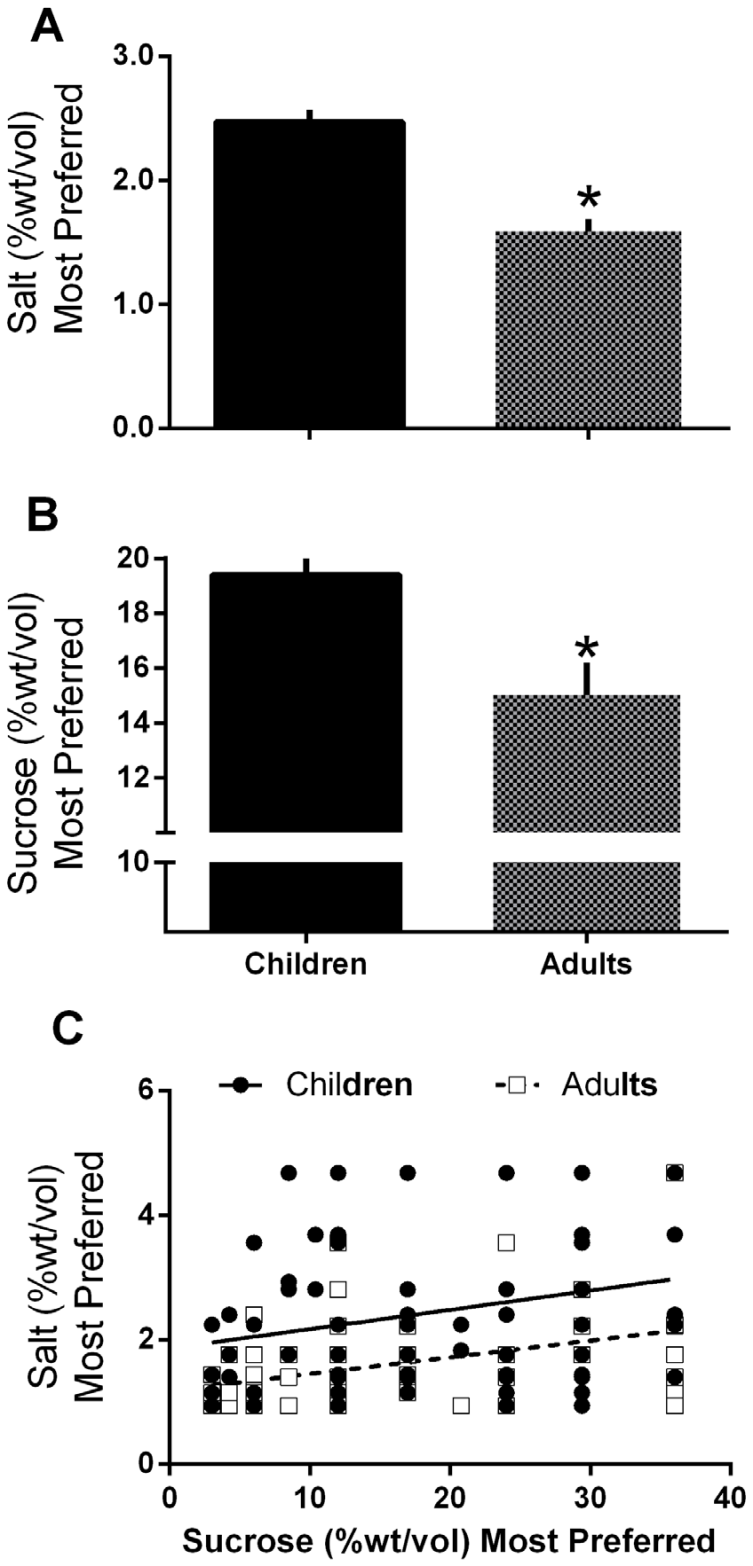
Most preferred levels of salty and sweet tastes for children and their mothers. (A and B) Children's most preferred levels of sweet (A) and salty (B) tastes were significantly higher than those of mothers (*p<0.01). (C) The most preferred levels of sweet and salty tastes were related both in children (p<0.05) and in mothers (p<0.001).

Children preferred higher concentrations of salt in the broth [F(1,169) = 30.71; p = 0.000); **Figure 1A**] and sucrose in water [F(1,174) = 7.93;p = 0.005), **Figure 1B**)] than did mothers (**Table 2**). Among individual children and adults, the most preferred levels of sweetness in water and in jellies were related [r(168) = 0.19; p = 0.01], as were the most preferred levels of saltiness in broth and in crackers [r(167) = 0.26; p = 0.001], regardless of age group. The most preferred concentrations of sucrose in water were significantly related to the most preferred levels of salt in broth both in children [r(94) = 0.25; p = 0.02)] and in mothers [r(73) = 0.42; p<0.001; **Figure 1C**]. Boys preferred more concentrated sucrose solutions than did girls [t(98) = −2.12, p = 0.04), but boys and girls did not differ in preference for saltiness of broth [t(94) = −0.40; p = 0.69].

**Table 2:** Univariate analysis of generation (mother vs. child) effects on sweet and salty taste preferences and genotype-related effects on sweet taste preferences.

	Least squares mean ± SEM, n	Statistics
***Most preferred level of salt (%w/vol) in broth***		
Children	2.47±0.11, n = 96	
Mothers	1.59±0.14, n = 75	Generation: F(1,169) = 30.71
***Most preferred level of sucrose (%w/vol) in water***		
Children	19.43±1.03, n = 100	Generation: F(1,174) = 7.93
Mothers	15.03±1.18, n = 76	p = 0.005
***Genotype effects on most preferred level of sucrose (%w/vol) in water*** a		
Children		Generation × genotype:
CC	20.84±1.74†, n = 31	F(2,138) = 4.48
CT	17.78±1.83†, n = 28	p = 0.01
TT	20.33±2.70†, n = 13	
Mothers		
CC	10.16±1.72*, n = 32	
CT	16.88±1.91†, n = 26	
TT	19.43±2.60†, n = 14	

a Post-hoc analysis; different symbols (*,†) denote genotype groups with sucrose preference values significantly different from each other. For siblings, one child was picked randomly for this analysis so that all children were unrelated.

**Figure 2:**
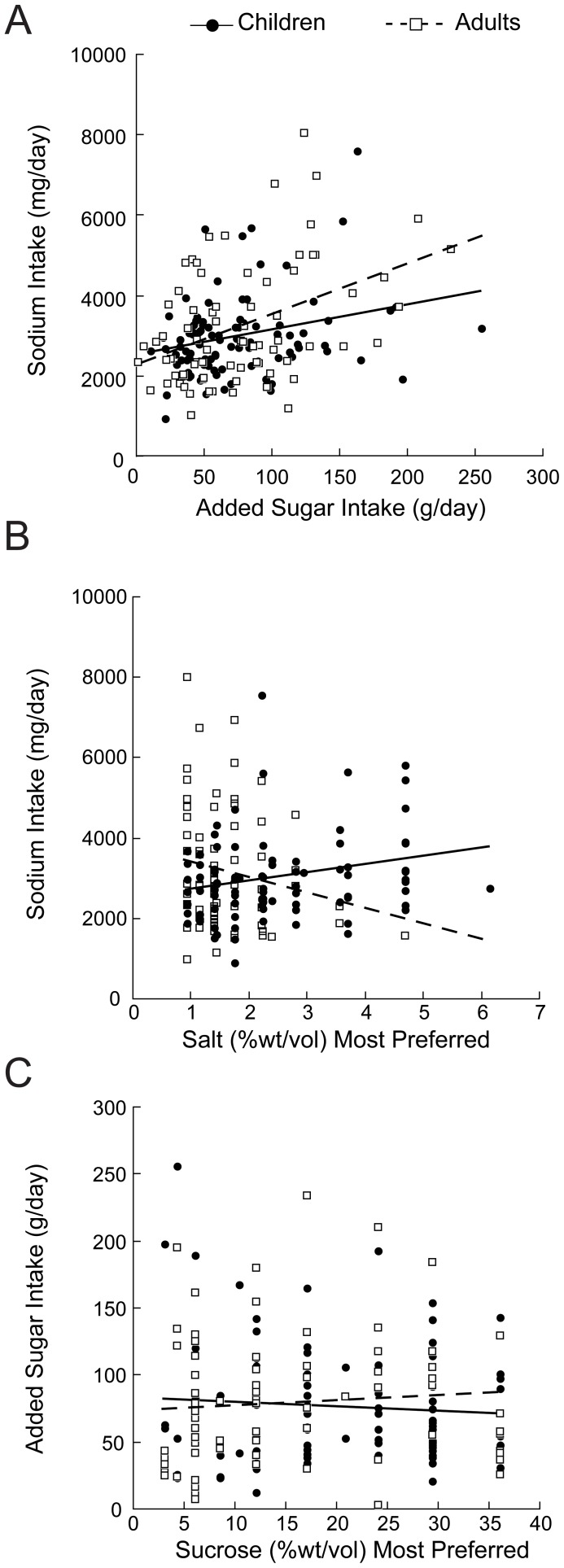
Correlation between salt and sugar intake and preferences. (A) Reported sugar and sodium intake correlated in children (p<0.01) and in mothers (p<0.001). (B) Daily sodium intake was associated with preferred salt level in broth in children (p<0.05) but not in mothers. (C) Daily added sugar intake was not related to preferred sucrose levels.

Reported dietary intakes of added sugars were significantly related to reported sodium intake in children [r(93) = 0.27; p = 0.007] and in mothers [r(72) = 0.42; p<0.001; **Figure 2A**]. The relationship between dietary added sugars and sodium remained significant with BMI or total caloric intake was a covariate in the analyses. There was concordance between children and mothers in intakes of dietary added sugar [r(92) = 0.23; p = 0.04] and dietary sodium [r(92) = 0.28; p = 0.009]. Average daily intakes of added sugar and sodium (**Table 1**) were above recommended levels [2], [32]. The more salt children preferred in broth, the more sodium in their diet [r(93) = 0.24; p = 0.02], but mothers showed the opposite tendency [r(73) = −0.20; p = 0.09; **Figure 2B** **]**. Daily added sugar intake was not correlated with most preferred concentration of sucrose in water in either age group (**Figure 2C**).

### Children's preferences, anthropometry, and a bone resorption marker

**Figure 3:**
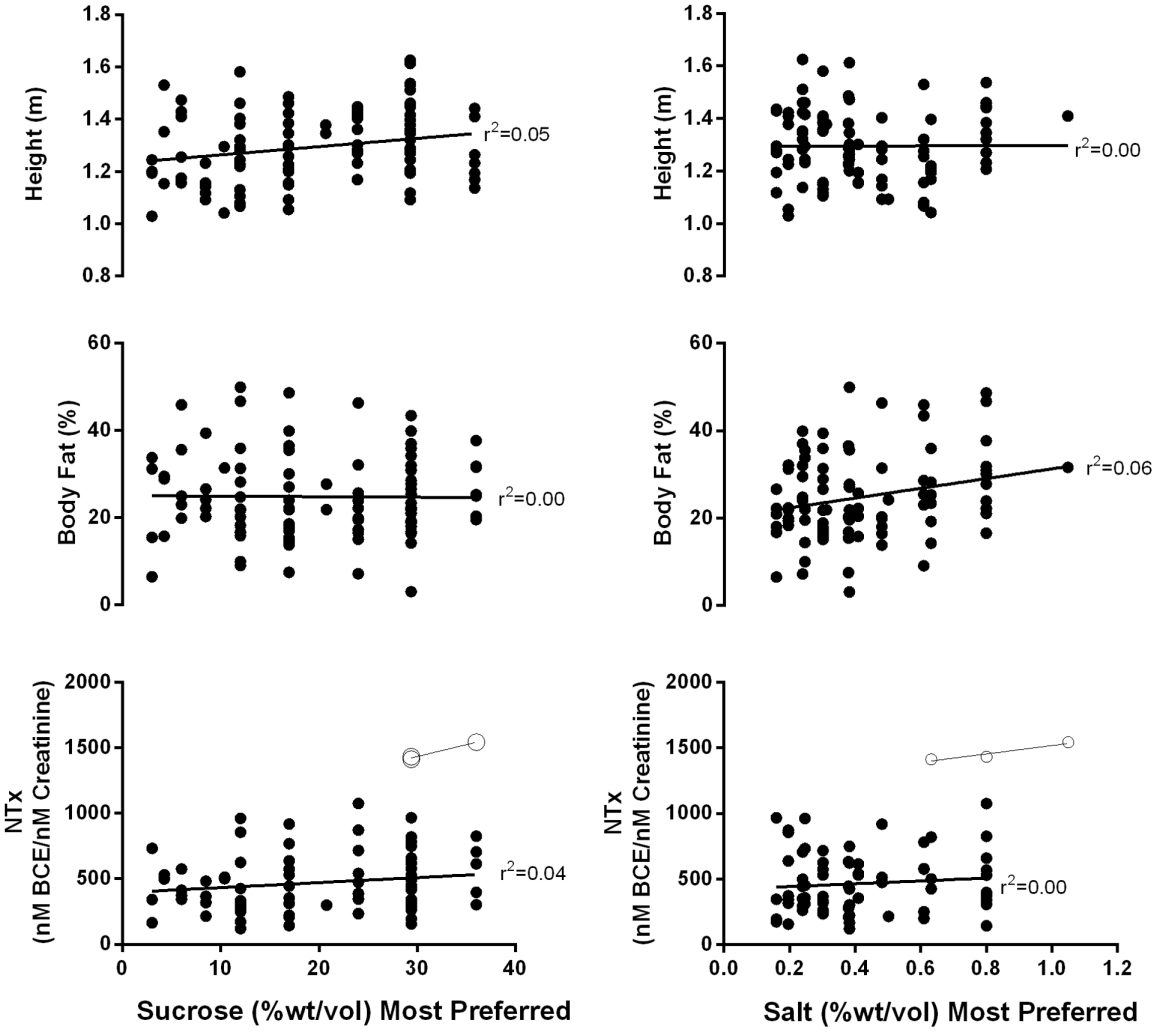
Associations between sweet and salty taste preferences and height, percent body fat, and NTx. Panels show associations between most preferred levels of sweet (left) and salty (right) tastes and height (top), percent body fat (middle), and NTx (bottom). Three outlying values of NTx (open data points) were removed for most analyses but are shown here for comparison.

The children differed markedly in body size and composition. Thirty of the children (29.7%) had a BMI at or above the 85th percentile; heights ranged from 1.00 to 1.63 m, and percent body fat ranged from 3.1% to 49.0%. NTx levels (mean ± SEM, 506.3±32.4 nM BCE/mM creatinine; range, 120–1,543 nM BCE/mM creatinine) were normally distributed and within the range reported previously for this age group [23]. However, three NTx values appeared to be outliers and so most analyses were conducted with as well as without these outliers, including the correlations below. Sweet and salty taste preference measures were related to some anthropometry measures: the most preferred sucrose concentration was positively correlated with height [r(98) = 0.23, p = 0.023] but not with NTx levels, body weight, or percent body fat; and the most preferred salt concentration was positively correlated with percent body fat [r(91) = 0.24, p = 0.02] but not with height, NTx levels, or body weight (**Figure 3**). Similar findings were found when the data were analyzed by grouping children based on sucrose preference value, but not when we grouped children based on median salty taste preference value. Children did not differ in age between the high and lower preference groups (**Table S1**).

Multivariate models were conducted to explore multiple factors that were related to preferences identified during the univariate analysis. We examined the relationship between race/ethnicity, maternal education and family income with salt and sugar preference but these variables lacked predictive value and were not considered in the statistical models (all p-values >0.20). When outliers of NTx values from three girls were included (**Table 3**, Model 1), sweet and salty taste preferences were related to this growth measure; however, when these extreme values were removed from the analysis, there was no effect of NTx (**Table 3**, **Figure 3**, **Model 2**). When the sweet and salty taste preferences were considered separately, with NTx outliers removed, the results were very similar to the correlational analyses: sucrose preference was related to height (**Table 3**, Model 3; effect size = 0.15), and salt preference, to percent body fat (**Model 4**; effect size = 0.08). Sex was a minor determinant of sweet but not salty taste preference; age alone was not a determinant of either preference in any model tested.

**Table 3:** Multivariate models.

Predictor		η^2^	*F*	Effect df	Error df	*p*
***Model 1: All growth and anthropometric variables; sucrose and salt preference as joint outcome measures***						
Sex		0.07	2.52	2	68	0.088
** Height (m)**		0.14	5.57	2	68	0.006
** NTx/creatinine**		0.12	4.74	2	68	0.012
** Percent body fat**		0.12	4.42	2	68	0.016
Age (years)		0.05	1.73	2	68	0.186
***Model 2: Same as Model 1 but with no NTx outliers***						
** Sex**		0.09	3.40	2	64	0.040
** Height (m)**		0.15	5.74	2	64	0.005
NTx/creatinine		0.01	0.19	2	64	0.826
**Percent body fat**		0.11	3.91	2	64	0.024
Age (years)		0.06	2.03	2	64	0.140
***Model 3: Same as Model 2 but with sucrose preference as only outcome measure***						
** Sex**		0.07	5.33	1	65	0.024
** Height (m)**		0.15	11.43	1	65	0.001
NTx/creatinine		0.00	0.29	1	65	0.588
Percent body fat		0.02	1.29	1	65	0.260
Age (years)		0.05	3.45	1	65	0.068
***Model 4: Same as Model 2 but with salt preference as only outcome measure***						
Sex		0.03	2.30	1	65	0.13
Height (m)		0.01	0.78	1	65	0.38
NTx/creatinine		0.00	0.14	1	65	0.71
**Percent body fat**		0.08	5.89	1	65	0.02
Age (years)		0.02	1.08	1	65	0.30
	

η^2^, proportion of variance accounted for; *F*, Fisher value; df, degrees of freedom. Only children with complete data for these variables were included in the analysis (e.g., n = 78 for Model 1). Variables shown in boldface made a significant contribution to the model.

### Sweet taste receptor genotypes

While there was no overall effect of genotype on most preferred level of sucrose in water, there was a significant interaction between age groups (mothers vs. children) and the *TAS1R3* sweet receptor gene rs35744813 variant on the most preferred level of sweetness (F(2,138) = 4.48, p = 0.013). Mothers, but not children, with no T alleles preferred a lower concentration of sucrose than did those with one or two T alleles (**Table 2** **; Figure S1**).

## Discussion

Because of the increasing concern of health risks associated with high intakes of salty and sweet foods by children, we examined whether salty and sweet taste preferences in children are related to each other and to markers of growth, as well as generational differences in sweet and salty taste preferences and their relationships to reported dietary intakes. We found that, among 5- to 10-year-olds and their mothers, children preferred higher concentrations of salt in broth and sucrose in water than did adults, and these preferences were significantly and positively correlated in both groups. The popular idea that children or adults have a sweet *or* salty preference is misleading – those that like very sweet foods more often also like very salty foods as well. These results confirm the first of the three predictions: the liking for the two prototypical tastes was concordant regardless of age and generalized to a variety of foods. Those children and adults who preferred higher concentrations of sucrose in water preferred higher concentrations of salt in broth, which in turn reflected liking for higher concentrations of salt and sugar in the more complex foods crackers and jellies, as well as reported dietary intake of sodium for children. Some have suggested that the positive association between dietary salt intake and sweetened beverage consumption among children and adolescents is due to sodium-induced thirst [33], [34]. The present findings suggest a more parsimonious explanation: a biological drive underlying the selection of these high-salt, high-sugar foods.

However, we found that sucrose preference did not correlate with reported sugar intake in children. Measuring real-world food intake from diet records has limitations, but this lack of congruence between preference and intake is not likely due to insensitive methods. We draw this conclusion because the daily intakes of added sugars (g/day) and sodium (mg/day) in the present study (**Table 1**) are remarkably consistent with intake data obtained from larger-scale epidemiological studies [4], [8]. Instead, this discrepancy may be due to the increasing use of non-nutritive sweeteners in the food supply [35], especially in foods geared for children. In other words, the intake of added sugars in the diet may not be a good proxy for the overall level of “sweetness” in their diets. In addition to concordance between levels of saltiness and sweetness most preferred, there was concordance in reported dietary intake among mothers and children for salt and sugars. Such concordance may be due to mothers regulating the types of foods proffered to children [36]. However, when given a choice, children preferred more intense sweetness and saltiness than did mothers, and perhaps parents exercise enough control over sugar consumption of children to moderate the relationship between preference and consumption.

The results also confirm the second of the three predictions: measures of growth and development are related to children's taste preferences, especially sweet taste. Sweet taste preference was closely tied to height, and salty taste preference was more closely tied to body fat. We confirmed the previously observed relationship between sweet taste preferences and NTx [17] in a younger aged group of children, but only if children with the most extreme values were included in the analysis. We note, however, that despite having extremes levels in reference to the other children, the NTx values were in normal range [23] and it may be that these values are ‘biologically relevant' and indicative of early puberty for these 3 girls, all of whom were preferring very high levels of sucrose in water and salt in broth. In multivariate analyses, children who were taller than their peers preferred a more concentrated solution of sucrose, and children with a higher percent body fat than their peers (a hallmark of accelerated maturation [19]) preferred a saltier-tasting broth. However, the latter relationship between body fat and salty taste preference was more tenuous and not statistically significant in all analyses. Height, percent body fat, NTx, and age were not related to the jelly or cracker preferences or to salt and sugar intake from diet records, possibly because (a) jelly and cracker tests are not as sensitive as measures of sucrose and salt preference, (b) 2-day diet records may not capture habitual diet, and/or (c) children's diet may be more regulated by parents than by children's likes and dislikes. While all these analyses should be interpreted with caution with small samples sizes, we favor the idea that preferences, at least for sweet tastes, may be driven by the demands of physical growth.

The popular notion that sweet taste preference is related to obesity was not observed in this study or in prior studies; unlike women, differences in the creaminess (fat content) of a pudding were not an important driver of preferences for children (14). For foods high in sweet and fat, sweet was the more attractive quality for children. There was, however, some indication of a relationship between obesity and salt preference. Given that many high-salt foods are also high in calories, it may be that salt preference in a food environment with many salty and high-calorie choices can affect adiposity. Or it could be that adiposity is a proxy measure for growth and development and is biologically allied with salt preference, the increase in adiposity and bone growth that occurs during puberty.

Although similar ages, boys tended to prefer sweeter solutions than did girls, but these effects were small. We found no difference between boys and girls in the level of salt most preferred, although a prior study in healthy adults found sex differences in salt liking [37]; whether such differences are due to differences in methodologies, age, and/or dietary patterns, especially those related to sodium intake [37], [38], is an important area for future research. The timing of growth differs between boys and girls, so a longitudinal study would be ideal to parse the effects of sex from the pattern of growth and their effects on taste preferences.

Inborn genetic differences partially accounted for individual differences in sweet preferences: in adults, the preferred concentration of sucrose was related to sweet receptor genotype, consistent with previous findings on sweet sensitivity [20] and preference [14]. We found no such relationship in children, indicating that developmental effects swamp genotype effects on sweet taste liking [14]. Future research should examine whether variation in other biological features of the taste system [39], [40] contributes to these age-related findings.

While collecting psychophysical data on taste can be complex [41], the results support methods to simplify data collection: preferences for simple sucrose and salt solutions can be used not only to predict preference for everyday foods but also to determine whether efforts to reduce dietary sodium affect taste preferences. In a landmark study [42], adult participants were fed a diet with substantial overall reduction (30–50%) in sodium content for 2–3 months and gradually developed a preference for foods with lower salt levels. Whether such strategies are effective for children is unknown. Although cross-sectional studies such as the current one have limitations, future research tracking parameters of growth, diet, and taste preferences is warranted to understand the driving forces underlying these taste changes (see also [6]) and to determine whether different strategies are needed to affect children's dietary intake of salts and sugars and preferences for these tastes during growth spurts.

## Conclusion

Because children naturally prefer higher levels of sweet and salty tastes than do adults, they are vulnerable to the modern diet, which differs from the diet of our past, when salt and sugars were once rare and expensive commodities. Having children eat diets low in sodium and added sugars requires a social, political, and economic food environment that supports and promotes this behavior change [43]. The present findings reveal that the struggle parents have in modifying their children's diets to comply with recommendations from medical authorities appears to also have a biological basis. Understanding the basic biology that drives the hedonic dimension of sweet and salty taste in children not only illustrates their vulnerability to the current food environment but also paves the way toward developing more insightful and informed strategies for promoting healthy eating that meet the particular needs of growing children.

## Supporting Information

Figure S1 **Plots of sweet preference for children and their mothers by** ***TAS1R3*** **genotype.** Gray bars are group means.
(TIF)

Table S1 **Univariate analyses of anthropometric measures based on grouping of sweet and salty taste preferences of children.**
(DOCX)
