# Supplementary figures and images for: Preferences for Salty and Sweet Tastes Are Elevated and Related to Each Other during Childhood
Source: PLoS One. 2014 Mar 17;9(3):e92201. doi: 10.1371/journal.pone.0092201 (PMC3956914; doi:10.1371/journal.pone.0092201)

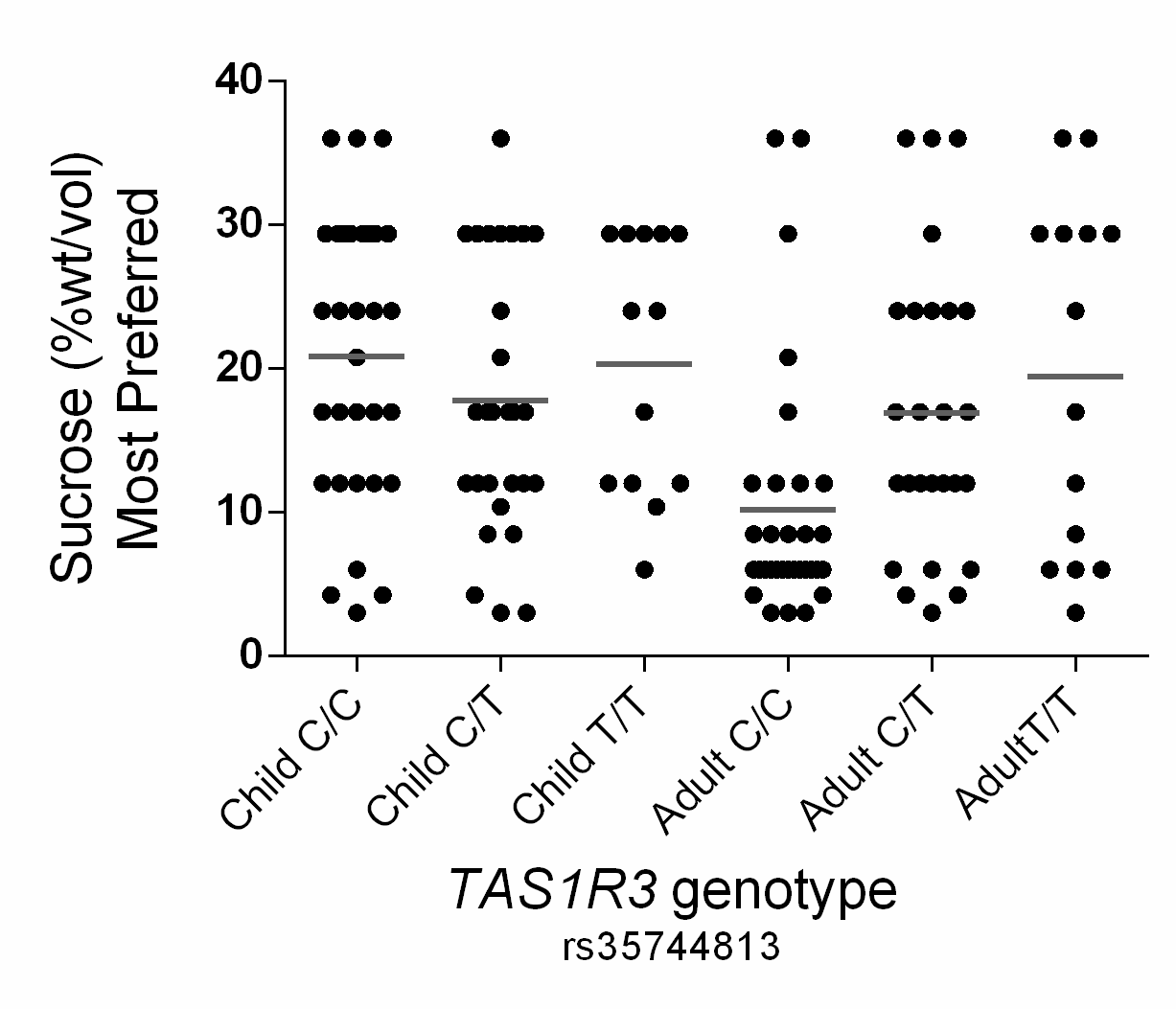

Supplement: Figure S1 — Plots of sweet preference for children and their mothers by TAS1R3 genotype. Gray bars are group means. (TIF) [file pone.0092201.s001.tif]
