# Supplementary material for: Preferences for Salty and Sweet Tastes Are Elevated and Related to Each Other during Childhood
Source: PLoS One. 2014 Mar 17;9(3):e92201. doi: 10.1371/journal.pone.0092201 (PMC3956914; doi:10.1371/journal.pone.0092201)
Supplement: Table S1 — Univariate analyses of anthropometric measures based on grouping of sweet and salty taste preferences of children. (DOCX) [file pone.0092201.s002.docx]

Supplemental Information

**Table S1.** Univariate analyses of anthropometric measures based on grouping of sweet and salty taste preferences of children

| **Grouping** | **Least squares mean ± SEM, n** | | **Statistic** |
| --- | --- | --- | --- |
| 1. ***Sweet Taste Preference Grouping*** | ***Group A: most preferred level of sucrose <19.4 %w/vol*** | ***Group B: most preferred level of sucrose >19.4 %w/vol*** |  |
| Height (m) | 1.26±0.02, n=53 | 1.33±0.02, n=47 | t(98)=-2.82; **p=0.006** |
| Weight (kg) | 28.19±1.44, n=53 | 31.88±1.53, n=47 | t(98)=-1.75; p=0.08 |
| BMI (m/kg^2^) | 17.23±0.48, n=53 | 17.48±0.48, n=47 | t(98)=-0.38; p=0.71 |
| Percent body fat | 25.15±1.39, n=49 | 24.50±1.42, n=47 | t(94)=0.33; p=0.74 |
| NTx/creatinine |  |  |  |
| All subjects | 438.75±43.64, n=42 | 581.04±45.88, n=38 | t(78)=-2.25, **p=0.027** |
| Minus outliers^b^ | 438.75±34.2, n=42 | 505.4±37.51, n=35 | t(75)=-1.31;p=0.19 |
| Age of children (years) | 7.54±0.26 | 8.14±0.26 | t(98)=-1.62, p=0.11 |
| 1. ***Salty Taste Preference Grouping*** | ***Group A: most preferred level of salt <2.23 %w/vol*** | ***Group B: most preferred level of salt >2.23 %w/vol*** |  |
| Height (m) | 1.31±0.02, n=43 | 1.28±0.02, n=53 | t(94)=0.81, p=0.42 |
| Weight (kg) | 29.98±1.64, n=43 | 30.37±1.48, n=53 | t(94)=-0.18, p=0.86 |
| BMI (m/kg^2^) | 16.98±0.50, n=43 | 17.81±0.45, n=53 | t(94)=-1.24, p=0.22 |
| Percent body fat | 23.69±1.51, n=42 | 25.72±1.37, n=51 | t(91)=1.00, p=0.32 |
| NTx/creatinine |  |  |  |
| All subjects | 459.29±50.71, n=33 | 537.08±43.43, n=45 | t(76)=1.17, p=0.24 |
| Minus outliers^b^ | 459.29±39.1, n=33 | 470.93±34.66, n=42 | t(73)=0.22, p=0.82 |
| Age of children (years) | 8.11±0.28, n=43 | 7.73±0.25, n=53 | t(94)=1.36, p=0.18 |

^a^All dependent measures were checked for normality. Groupings are based on median split. n=number of subjects. Significant values are shown in boldface.

^b^Analyses were recalculated after eliminating the three subjects with values >1,200 (although within normal range and normal distribution).
